# Epigenetic quantification of immunosenescent CD8^+^ TEMRA cells in human blood

**DOI:** 10.1111/acel.13607

**Published:** 2022-04-09

**Authors:** Ahto Salumets, Liina Tserel, Anna P. Rumm, Lehte Türk, Külli Kingo, Kai Saks, Astrid Oras, Raivo Uibo, Riin Tamm, Hedi Peterson, Kai Kisand, Pärt Peterson

**Affiliations:** ^1^ 37546 Molecular Pathology Institute of Biomedicine and Translational Medicine University of Tartu Tartu Estonia; ^2^ 37546 Institute of Computer Science University of Tartu Tartu Estonia; ^3^ 37546 Department of Dermatology and Venereology Institute of Clinical Medicine University of Tartu Tartu Estonia; ^4^ 37544 Clinic of Dermatology Tartu University Hospital Tartu Estonia; ^5^ 37546 Department of Internal Medicine Institute of Clinical Medicine University of Tartu Tartu Estonia; ^6^ 37546 Department of Immunology Institute of Biomedicine and Translational Medicine University of Tartu Tartu Estonia; ^7^ Laboratory of Immune Analysis United Laboratories Tartu University Hospital Tartu Estonia

**Keywords:** biomarkers, CD8^+^ T‐cells, CMV, epigenetics, human aging, inflammation

## Abstract

Age‐related changes in human T‐cell populations are important contributors to immunosenescence. In particular, terminally differentiated CD8^+^ effector memory CD45RA^+^ TEMRA cells and their subsets have characteristics of cellular senescence, accumulate in older individuals, and are increased in age‐related chronic inflammatory diseases. In a detailed T‐cell profiling among individuals over 65 years of age, we found a high interindividual variation among CD8^+^ TEMRA populations. CD8^+^ TEMRA proportions correlated positively with cytomegalovirus (CMV) antibody levels, however, not with the chronological age. In the analysis of over 90 inflammation proteins, we identified plasma TRANCE/RANKL levels to associate with several differentiated T‐cell populations, including CD8^+^ TEMRA and its CD28^−^ subsets. Given the strong potential of CD8^+^ TEMRA cells as a biomarker for immunosenescence, we used deep‐amplicon bisulfite sequencing to match their frequencies in flow cytometry with CpG site methylation levels and developed a computational model to predict CD8^+^ TEMRA cell proportions from whole blood genomic DNA. Our findings confirm the association of CD8^+^ TEMRA and its subsets with CMV infection and provide a novel tool for their high throughput epigenetic quantification as a biomarker of immunosenescence.

## Graphical abstract

We studied old individuals over 65 years of age. We studied their CD4^+^ and CD8^+^ T cell subpopulations, quantified the CMV‐specific antibodies, inflammation‐associated proteins, and DNA methylation levels of selected CpGs. Based on our data we developed an epigenetic model to estimate CD8^+^ TEMRA cell levels in whole blood. The figure was created with BioRender.com.

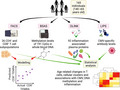

## 1 INTRODUCTION

Deterioration of T‐cell function has a central role in the age‐related impairment of immune responses associated with an increased risk of infections and chronic diseases, and poor vaccine efficacy (Nikolich‐Žugich, 2018). Predominant changes in the T‐cell compartment are related to loss of thymic output, resulting in lower production of new antigen‐naive T‐cells. In addition, T‐cell subpopulations with late differentiation and effector functions increase in old individuals (Mittelbrunn & Kroemer, 2021); (Elyahu & Monsonego, 2021).

With age, the human naïve CD4^+^ T‐cell population relies more on the homeostatic proliferation of existing T‐cell clones rather than de novo generation of new ones from the thymus (Goronzy & Weyand, 2019). The cell division of naive CD4^+^ T‐cells depends on homeostatic cytokine IL‐7 and they retain higher expression of IL‐2 receptor CD25, enabling them to receive sufficient survival and proliferation signals for cellular maintenance (Sprent & Surh, 2011). Nevertheless, the progressive thymic involution and decreased naive T‐cell turnover in the periphery results in the decline of naïve CD4^+^ T‐cells and increase of effector memory (EM) cells with age (Mold et al., 2019).

The homeostatic proliferation is less efficient in maintaining the naïve CD8^+^ T‐cell population, and the numbers of differentiated, exhausted, and senescent CD8^+^ effector and memory cells accumulate faster than in the CD4^+^ population (Mold et al., 2019). The population of terminal effector memory CCR7^−^CD45RA^+^ (TEMRA) T‐cells and its subsets (Appay et al., 2002), characterized by increased sensitivity to innate signals, a decline in T‐cell receptor (TCR) dependent activation, and lower TCR clonal diversity, is particularly prominent among old individuals (Henson et al., 2012). The loss of membrane receptors CD28, CD27, and CD127, re‐expression of CD45RA, and expression of exhaustion marker PD1^+^ or senescence marker CD57 in CD8^+^ TEMRA cell populations are likely caused by several factors, of which cumulative antigenic load induced by chronic viral infections is considered prevalent.

Frequent reactivation of latent cytomegalovirus (CMV) repetitively stimulates CD8^+^ T‐cells, increasing their population size (Pawelec, 2014; Sylwester et al., 2005). The repetitive activation by CMV is considered as the main driving force of inflammaging (Franceschi et al., 2000), a low‐grade chronic inflammatory state associated with the release of pro‐inflammatory cytokines (Aiello et al., 2017; Akbar et al., 2016). Large CMV‐specific responses observed in older people correlate with the presence of CMV antibodies and are also implicated in T cell “memory inflation” (Akbar et al., 2016; Huang et al., 2019). Although the role of CMV in clinical outcomes and CD8^+^ T‐cell alterations are not fully understood, higher proportions of CD8^+^ TEMRA cells often indicate adverse health consequences (Pawelec et al., 2014).

The high levels of CD8^+^ TEMRA cells correlate positively with CMV serostatus (Souquette et al., 2017; Wertheimer et al., 2014) and with age‐related chronic inflammation and several comorbidities (Boßlau et al., 2021; Chiu et al., 2018, 2020; Jacquemont et al., 2020; Spyridopoulos et al., 2016; Yang et al., 2018; Yu et al., 2016). Their quantification may serve as a potential tool to measure T‐cell immunosenescence, however, this has so far relied on cell counting by flow cytometry that requires a sufficient sample of intact leukocytes from well‐preserved blood and time‐critical cytometry analysis, which is not always feasible for various medical applications.

Herein, we conducted a broad T‐cell profiling in older individuals and correlated the results with their CMV antibody and over 90 inflammatory marker levels. We performed deep‐amplicon bisulfite sequencing of preselected CD8^+^ specific CpG sites and developed an epigenetic cell quantification model for CD8^+^ TEMRA cells. The epigenetic quantification could provide a high throughput biomarker for the stratification of immunosenescence and to monitor immune health status in patients with chronic inflammatory diseases.

## 2 RESULTS

Aging is characterized by prominent changes among T lymphocytes and other immune cell populations. Using moving average, we confirmed previously reported increase in monocyte and neutrophil numbers, reduction in CD4^+^ T‐cells, and increase in CD8^+^ TEMRA cells in individuals ranging from 4 to 96‐year‐old (Figure S1A–C; Table S2). In particular, we found a steady increase of CD8^+^ TEMRA cells over the years with gradient rise after 50 years of age and emergence of CD4^+^ TEMRA cells in the same period. This trend indicated that the increase in TEMRA populations becomes prominent at the sixth decade of life, prompting detailed analysis of T‐cell populations in older individuals.

### 2.1 Differentiated CD8^+^ T‐cells are prevalent and have high degree of interindividual variation

**FIGURE 1:**
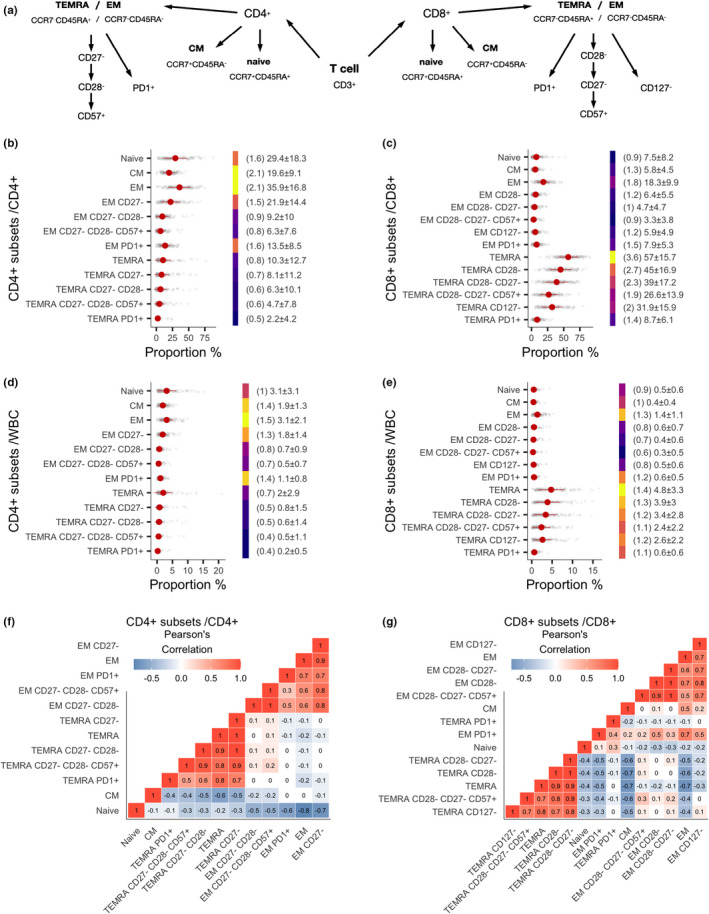
Increased proportions and high interindividual variability of CD4^+^ and CD8^+^ T‐cell subsets. (a) Schematic picture of studied CD4^+^ and CD8^+^ T‐cell populations. (b–e) Relative sizes of CD4^+^ and CD8^+^ T‐cell subsets among CD4^+^ (b) and CD8^+^ (c) compartments and among whole blood cells (WBC) (d for CD4^+^ subsets, e for CD8^+^ subsets). Red point shows the mean and adjacent line a standard deviation. The color bar shows signal‐to‐noise ratio (SNR) calculated as mean/SD with brighter color denoting higher SNR value (in brackets). In addition, mean and standard deviation are written next to each measurement. The two heatmaps (f and g) are based on correlation matrices that contain pairwise Pearson's correlation coefficients in CD4^+^ and CD8^+^ T‐cell subsets, respectively

We investigated the proportion and interindividual variation of T‐cell populations among old individuals. For this, we studied 26 CD4^+^ and CD8^+^ T‐cell subpopulations by flow cytometry in a cohort of 140 persons with an age range from 65 to 96 years and a female‐male ratio of 3 to 1 (Figure 1a, Table 1).

**TABLE 1:** Gender, age, disease, and CMV seropositivity of the old individuals' cohort

	*n*	Proportion	SD
Total	140		
Gender			
F	105	0.75	
M	35	0.25	
Age			
65–69	30	0.21	1.44
70–74	28	0.2	1.33
75–79	39	0.28	1.47
80–84	30	0.21	1.36
85–89	8	0.06	1.55
90–94	3	0.02	1
95–99	2	0.01	0.71
Disease (ICD0 code)			
Hypertension (I10)	105	0.75	
Type 2 diabetes (E11)	34	0.24	
Kidney disease	20	0.14	
Chronic (N18)	19	0.14	
Acute (N17)	1	0.01	
CMV			
CMV−	10	0.07	
CMV+	103	0.74	
NA	27	0.19	

The CD4^+^ and CD8^+^ T‐cells were divided based on their expression of CD45RA and CCR7 into naive (CD45RA^+^ CCR7^+^), central memory (CM; CD45RA^−^ CCR7+), effector memory (EM; CD45RA^−^ CCR7^−^), and terminally differentiated effector memory (TEMRA; CD45RA^+^ CCR7^−^) cells. Among EM and TEMRA populations, we studied four CD4^+^ subsets (a) CD27^−^, (b) CD27^−^ CD28^−^, (c) CD27^−^ CD28^−^ CD57^+^ and (d) PD1^+^, and five CD8^+^ subsets (a) CD28^−^, (b) CD28^−^ CD27^−^, (c) CD28^−^ CD27^−^ CD57^+^, (d) CD127^−^, and (e) PD1^+^. It should be noted that among CD4^+^ T‐cell compartment, the effector cells first downregulate CD27 and later CD28 marker, whereas this is opposite within CD8^+^ compartment. Thus, among the CD4^+^ T‐cell population CD27‐ cells are parent population for CD27^−^ CD28^−^, and these in turn are parent population for CD27^−^ CD28^−^ CD57^+^. In contrast, among the CD8^+^ T‐cell population CD28^−^ cells are parent population for CD28^−^ CD27^−^ and these are in turn a parent population for CD28^−^ CD27^−^ CD57^+^. In contrast, the CD4^+^ and CD8^+^ EM and TEMRA PD1^+^ cells are separate subsets, as well as the CD8^+^ T‐cell subset that is negative for CD127 (IL7RA).

In the CD4^+^ compartment, the most prevalent subtypes were EM and naïve cells, on average 35.5% and 29.1%, respectively (Figure 1b). The majority (60.6%) of CD4^+^ EM cells were negative for the CD27 marker, and 13.3% had an exhaustion marker PD1 on their surface. The proportion of CD4^+^ TEMRA cells was relatively low, around 10%. From all blood cells, both CD4^+^ EM and naive cells formed on average 3.1% (Figure 1d). As the variation within the cellular compartments was in correlation with their size, we used signal‐to‐noise ratio (SNR) to pinpoint the interindividual variation in the cell populations, by dividing each cell population mean with its corresponding standard deviation. According to the spread of the data and SNR, the central memory (CM), naive and EM populations varied most among CD4^+^ T‐cells (Figure 1b and d).

Among CD8^+^ T‐cells, differentiated TEMRA cell subpopulations were most prevalent (Figure 1c and e). The proportion of CD8^+^ TEMRAs reached on average nearly 60% of all CD8^+^ T‐cells, and it was represented as a major cell type among all T‐cells and 4.8% of whole blood cells (WBCs; Figure 1e). The CD8^+^ TEMRA cells were dominated by age‐associated CD28^−^ or CD27^−^ and CD28^−^ subpopulations, of which many were positive for CD57, a senescence marker for T‐cells. We also found high inter‐individual variation among CD8^+^ TEMRA subpopulations, including CD28^−^, CD28^−^CD27^−^, CD127^−^, and PD1^+^ subsets (Figure 1c and e). Furthermore, in addition to their high numbers, the CD8^+^ TEMRA populations had the highest inter‐individual variability of all T‐cells in old individuals (Figure 1e). The data on mean values and inter‐individual variability of all studied T‐cells is in Table S1.

The subsets of EM, CM, and TEMRA cells correlated highly within their families, except PD1^+^CD8^+^ TEMRA that formed a separate cluster from other CD8^+^ TEMRA (Figure 1f and g). The CD4^+^ EM cell populations were in positive correlation with CD4^+^ and CD8^+^ TEMRA cell subsets and formed a joint cluster in a correlation matrix whereas, interestingly, CD8^+^ EM cells clustered together with CD4^+^ and CD8^+^ CM T‐cells (Figure S2). The CD4^+^ and CD8^+^ TEMRA cells were also in negative correlation with corresponding naive T‐cells.

**FIGURE 2:**
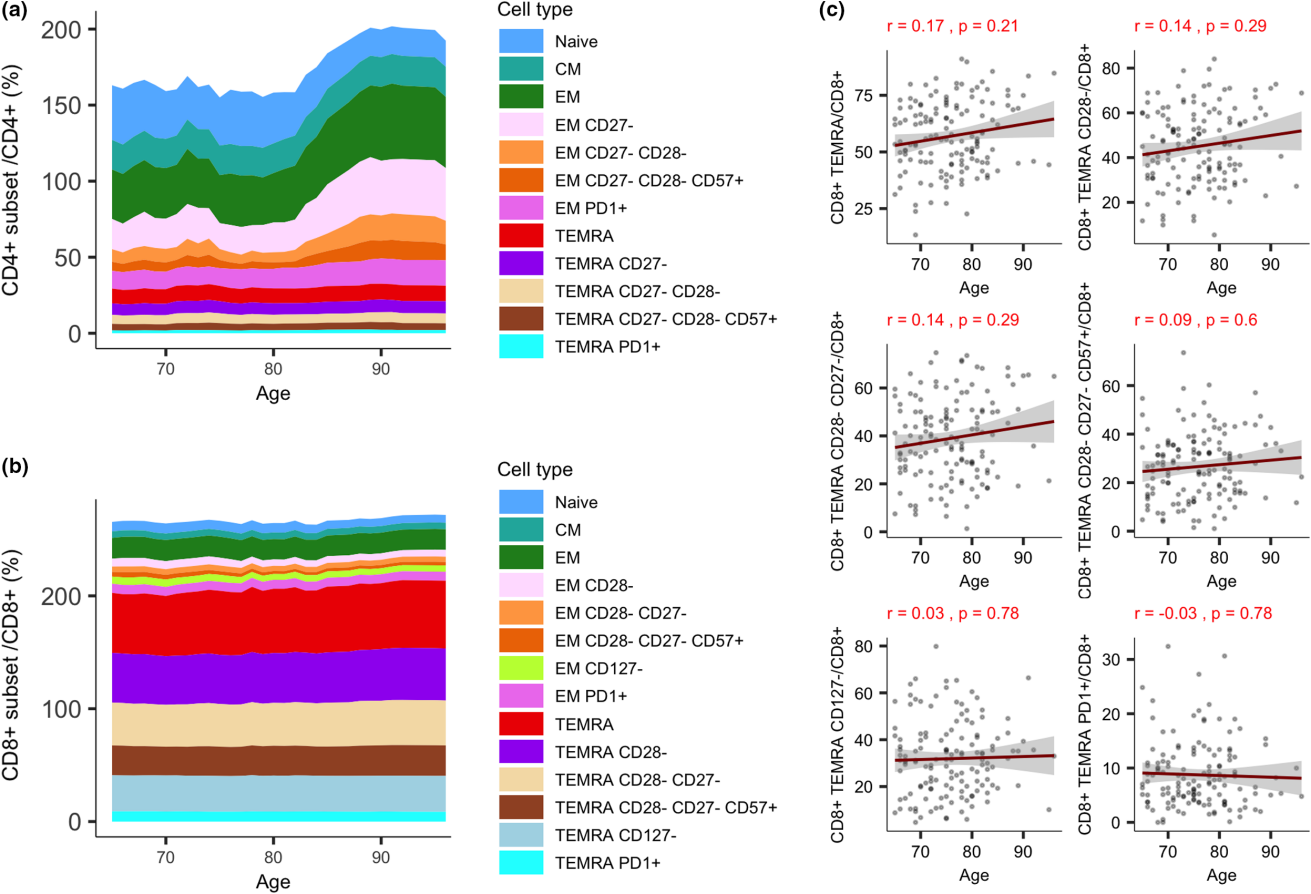
T‐cell subset dynamics in old individuals. The dynamics of CD4^+^ (a) and CD8^+^ (b) T‐cell subset sizes in old age (≥65 years), respectively, via moving average. (c) Scatterplots of CD4^+^ and CD8^+^ T‐cell subset changes in old age. Scatterplots report Pearson's correlation coefficient and an adjusted *p*‐value

An analysis of T‐cell population dynamics after the age of 65 years showed no significant changes (Table S3), although we saw a trend indicating a decline in CD4^+^ naive T‐cells and increase in CD4^+^ EM cells lacking CD27 marker after 80 years of age (Figure 2a), suggesting a continuous shift between CD4^+^ naive and effector cell populations. In contrast, the moving average in CD8^+^ cells was very stable (Figure 2b). Although both CD4^+^ and CD8^+^ TEMRA cells are considered as age‐related cell types, after 65 years their levels do not correlate with age (Figure 2c, Figure S3), implying that other factors are at play. In addition, since the study group expectedly had a diverse set of chronic diseases (e.g., hypertension, cardiovascular disease, type 2 diabetes [T2D], and chronic kidney disease), we did not find any significant association between cellular levels and those phenotypes (Figures S4 and S5, Tables S4 and S5).

### 2.2 Antibody responses to CMV correlate with the proportion of CD8^+^ TEMRA cells

**FIGURE 3:**
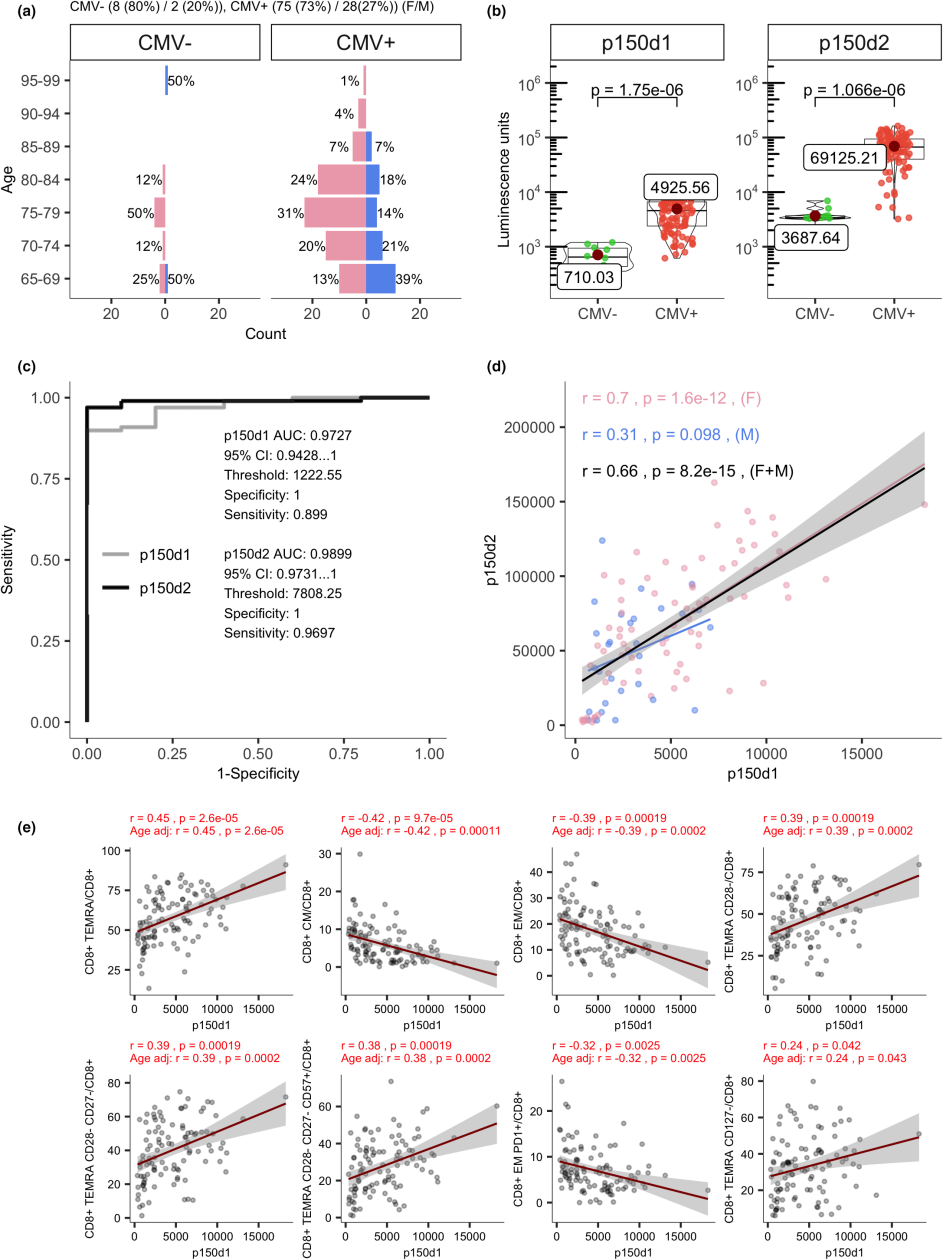
CMV‐specific antibody level correlations with T‐cell subsets in old individuals. (a) Age‐gender distribution of CMV positive and negative individuals. (b) The levels of anti‐p150d1 and p50d2 antibodies in CMV positive and negative individuals are shown as luminescence units (LU) of luciferase enzyme activity as boxplots. (c) ROC curve for the p150d1 and p150d2 fragments’ LIPS analysis shows the classification performance by dividing individuals into CMV positives and negatives. (d) Correlation between antibody levels to p150d1 and p150d2 fragments in LIPS measurements. (e) Correlation between p150d1 specific LIPS results and T‐cell subset proportions in flow cytometry shown together with and without age‐adjusted Pearson's correlation coefficient and adjusted *p*‐values

We next studied CD8^+^ TEMRA cells and their relation to the immune response to CMV as its chronic infection has been linked to immunosenescence (Aiello et al., 2017). To this end, we applied CMV antibody‐specific ELISA and found the majority of individuals over 65 years (91.2%) to be positive for CMV antibodies (Figure 3a). Because the CMV ELISA is not optimal for the quantification of CMV antibodies, we developed a LIPS (luciferase‐based immunoprecipitation system) method using NanoLuc enzyme recombinantly tagged to two highly immunogenic CMV tegument pp150 protein (Tomtishen, 2012) fragments (p150d1 and p150d2) to measure the antibody levels specific to CMV. LIPS has several advantages over ELISA as it uses recombinant target antigens in native conformation. LIPS output also ranges over multiple orders of magnitude making it suitable to monitor quantitative antibody levels. The LIPS with CMV p150 fragments was highly specific and able to measure anti‐CMV antibody levels with a broad quantitative range (Figure 3b). We tested its sensitivity and specificity in ROC analysis, which showed an excellent separability with AUC over 0.97 for fragment p150d1 (AUC 0.973) and p150d2 (AUC 0.990) when compared with ELISA results (Figure 3c). The antibody levels to CMV p150d1 and p150d2 fragments were in strong correlation (r = 0.66, Figure 3d), which was higher among females (r = 0.7 vs. r = 0.3) as a relatively small number of males had the antibodies with high levels.

We then compared the CMV p150 antibody levels with T lymphocyte populations (Figure 3e; Tables S6 and S7, and for both sexes separately Figure S6, Tables S8 and S9). Antibodies to p150d1 fragment correlated positively with CD8^+^ TEMRA cells and its subpopulations (CD28^−^, CD27^−^CD28^−^, CD57^+^). A similar trend was present between CD8^+^ TEMRA proportions and antibodies to the p150d2 fragment, albeit the correlations were weaker. In agreement with previous analyses, a negative correlation was present between CMV antibodies and CD8^+^ CM and EM cells.

### 2.3 CD8^+^ TEMRA cell associations with plasma inflammatory proteins

**FIGURE 4:**
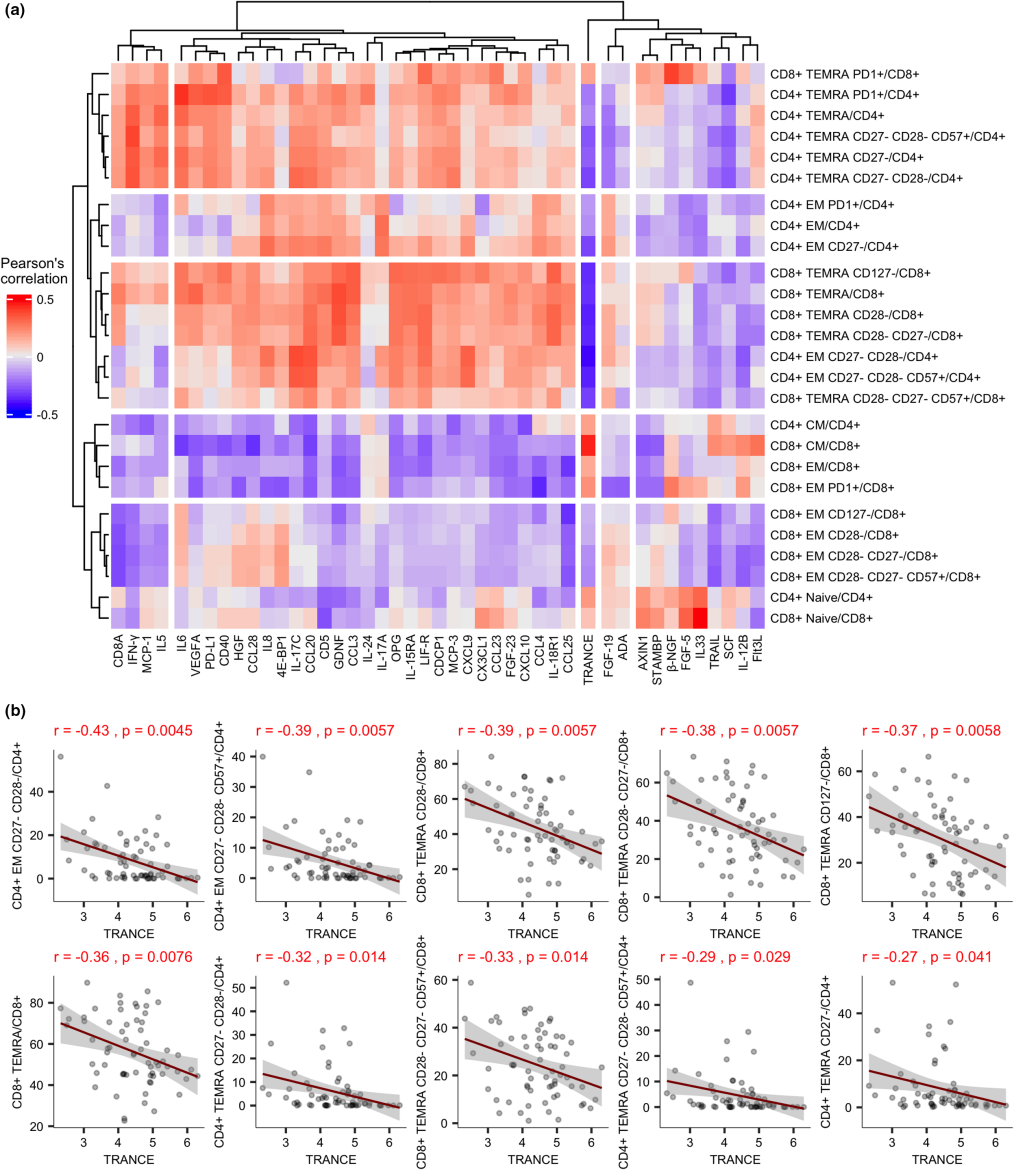
Plasma inflammation markers correlations with T‐cell subsets in old individuals. (a) Correlation between CD4^+^ and CD8^+^ T‐cell subset proportions and plasma inflammation markers measured by proximity extension profiling and shown as a clustered heatmap. (b) Top 10 correlations of TRANCE with the proportions of CD4^+^ and CD8^+^ T‐cell subsets. The inflammatory protein levels are shown as normalized protein expression (NPX) values, a metric that is on a log_2_ scale and where a higher value indicates a higher protein level. The Pearson's correlation coefficient and adjusted *p*‐value for each correlation are shown

Regarding the critical role of inflammation in T‐cell immunosenescence, we undertook a targeted proteomic analysis of 92 individual soluble inflammation‐associated proteins and calculated their corresponding correlations with CD4^+^ and CD8^+^ T‐cell populations in the same cohort of old individuals (Figure 4a, Table S10). The most prominent correlations in the clustered matrix of Olink proximity extension assay (PEA) measurements and T‐cells indicated the association of TRANCE protein with TEMRA cells (Figure 4a). TRANCE, also known as RANKL, is a TNF family member known for its function in bone remodeling, lymph node formation, and differentiation of thymic epithelial cells (Hanada et al., 2011). We took the two clusters containing CD4^+^ and CD8^+^ TEMRA cells together with CD27^−^ CD28^−^ and CD27^−^ CD28^−^ CD57^+^ CD4^+^ EM cells for detailed analysis of their correlations with TRANCE/RANKL (Figure 4b, Table S11). Overall, we saw a moderate negative correlation between TRANCE/RANKL and CD8^+^ TEMRA and its CD28^−^ and CD27^−^ CD28^−^, CD27^−^ CD28^−^ CD57^+^ and CD127^+^ subpopulations. Furthermore, it correlated negatively with highly differentiated CD27^−^CD28^−^ and senescent CD57^+^ EM CD4^+^ T‐cells, and a weaker negative association was also present with CD4^+^ TEMRA CD27^−^ and CD27^−^ CD28^−^ populations.

### 2.4 CD8^+^ TEMRA cell‐specific CpG methylation sites

Given its association with age‐related chronic inflammatory diseases and CMV infection, CD8^+^ TEMRA cell proportion could be regarded as a biomarker for immune senescence. Therefore, we aimed to create a computational model, based on epigenetic differences, to predict CD8^+^ TEMRA cell proportion in the whole blood. We focused on epigenetic quantification on WBC level as the peripheral blood is a common source of human genomic DNA.

Earlier age‐related DNA methylation profiling with Illumina HumanMethylation450 array enabled us to identify multiple candidate CpG sites in genes with differential methylation and expression within CD4^+^ and CD8^+^ T‐cell populations from old individuals (Tserel et al., 2015). From this dataset, we selected 191 CpG sites for site‐specific DNA methylation screening (Table S12), of which many were close to the genes expressed in differentiated T‐cells. We used bisulfite deep‐amplicon sequencing of the samples of 165 individuals to identify specific DNA methylation levels in those CpG sites using whole blood as a source of genomic DNA.

We next correlated the T‐cells proportions from flow cytometry analysis with the CpG site methylation levels (Table S13) to develop an epigenetic model for the estimation of the CD3^+^, CD8^+^, and CD8^+^ TEMRA frequencies within peripheral blood. Because the methylation and flow cytometry analyses reflect the proportions of T‐cell populations in the same tissue, we could assume a linear relationship between the methylation level of the cell‐specific CpG site and the fraction of a particular cellular subset in the blood. The methylation levels of multiple CpG sites clustered together within T‐cells, which differed from NK cells and neutrophils; and differential clustering was also present between CD4^+^, CD8^+^, and CD8^+^ TEMRA cells (Figure S7). Furthermore, all CD8^+^ TEMRA subsets (including CD28^−^, CD27^−^CD28^−^, CD27^−^CD28^−^ CD57^+^, and CD127^−^ populations), except CD8^+^PD1^+^ TEMRA, formed a distinct separate cluster from CD4^+^ TEMRA, naive CD8^+^, CD8^+^ EM, and CD8^+^ CM T‐cells, indicating their cell‐type‐specific DNA methylation pattern (Figure S8).

**FIGURE 5:**
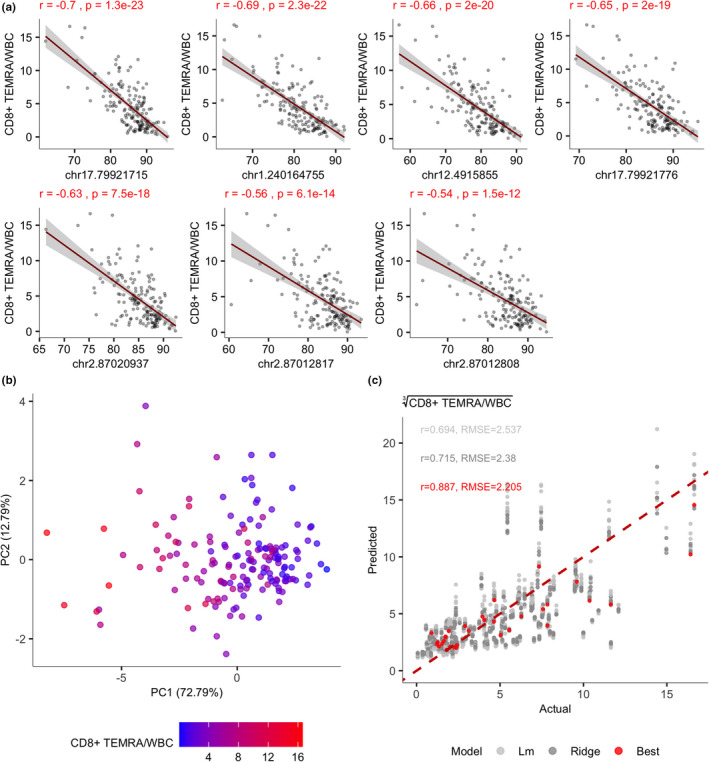
CD8^+^ TEMRA associations with methylations levels of selected CpG sites. (a) The correlations between methylation levels of CpG sites that were incorporated into the prediction model and CD8^+^ TEMRA cell proportions in WBC. (b) PCA calculated on the methylation levels of those 7 CpG sites in (a) and colored according to the level of percentages of CD8^+^ TEMRA/WBC. (c) The accuracy of the final model in red together with predictions of models that were built on resampled training dataset using linear (light gray) and ridge (dark gray) regression models. Those illustrate the variability caused by selecting different training and test set

Before the identification of the core set of CpG sites of interest, we reduced the number of CpG sites as it exceeded the number of observations. To this end, we applied several feature selection methods (further described in methods) to identify the most informative CpG sites for CD3^+^, CD8^+^, and CD8^+^ TEMRA cells. After this, we selected 12 CpG sites for CD3^+^ (Figure S9A), 5 CpGs for CD8^+^ (Figure S9B), and 7 CpGs for CD8^+^ TEMRA (Figure 5a) into the modeling task, with correlations ranging from moderate to strong (|r| = 0.42…0.70).

The 4 out of 7 CpG sites selected for predicting CD8^+^ TEMRA cells were located close to genes expressed in T‐cells: CD8A (chr2:87012808, chr2:87012817, chr2:87020937) and GALNT8/KCNA6 (chr12:4915855) genes. However, the highest correlations with CD8^+^ TEMRA cells of those CpG sites were present with chr1:240164755 on chromosome 1 and chr17:79921715 on chromosome 17, near to FMN2 and NOTUM genes, respectively (Figure 5a). The seven sites also correlated with the parent CD8^+^ T‐cell population; however, five of them had weaker correlations and only CpGs close to CD8A had comparable coefficients to CD8^+^ TEMRA sites. Altogether, those CpG sites convey information about CD8^+^ TEMRA levels as illustrated by a coloring on PCA plot (Figure 5b).

The models were built using 5‐fold cross‐validation altogether on 125 training instances. The random forest algorithm showed the best performance in the prediction of CD3^+^ cell proportion, while ridge regression was superior for predicting CD8^+^ and CD8^+^ TEMRA proportions. We validated these models on a test set containing 28 samples and measured the correlation and root mean squared distance (RMSE) between actual and predicted values. We obtained the most accurate model for CD3^+^ T‐cells (r = 0.915, RMSE 3.1) (Figure S9C). Likewise, the models for CD8^+^ T‐cells (Figure S9C) and CD8^+^ TEMRAs (Figure 5c) showed a closely similar correlation with their actual values (r = 0.877, RMSE = 2.1 and r = 0.887, RMSE = 2.2, respectively). Due to the limited sample size and high variability of target measurements, we confirmed the usefulness of our selected features by additionally sampling a new training and test set from the entire dataset multiple times and automatically built linear regression models using the same features. This further supported the suitability of the selected CpG sites in the prediction of the cellular compartments since even then the observed correlation between predicted and actual values remained strong (r > 0.6) (Figure 5c, Figure S9C, gray points).

## 3 DISCUSSION

Accumulating in old individuals, CD8^+^ TEMRA cells, which are characterized by their high cytotoxicity, low proliferation, and sensitivity to apoptosis, have been associated with excess inflammation and several chronic inflammatory conditions (Lanna et al., 2017) (Aiello et al., 2017; Yang et al., 2018).

We here confirmed the findings showing an increase of CD8^+^ TEMRA cells with age, in particular, a steep rise after 50 years of age together with the increase of CD4^+^ TEMRA cells. Both CD4^+^ and CD8^+^ senescent T‐cells lose the expression of the costimulatory CD28 and CD27 molecules and upregulate the expression of terminal‐differentiation markers, such as CD57, that has been used to identify senescent T‐cells (Akbar et al., 2016; Mittelbrunn & Kroemer, 2021). CD8^+^ TEMRA cells accumulate faster and our results highlight the high variability among its subsets in old individuals. Nevertheless, despite high interindividual variability, the CD8^+^ TEMRA subpopulations did not increase with the chronological age after 65 years. This suggests that age itself has no significant impact on differentiated T‐cell variability in old individuals and that the interindividual variability among CD8^+^ TEMRA subsets is more influenced by lifelong determinants such as chronic metabolic diseases and chronic virus infections.

As CMV infection has been associated with T‐cell immunosenescence and repertoire oligoclonality, we correlated CD8^+^ TEMRA cells to CMV antibodies and found their positive correlation with CD8^+^ TEMRA cells and its subpopulations (CD28^−^, CD27^−^CD28^−^, and CD57^+^). The chronic CMV infection is known to drive repeated CD8^+^ T‐cell stimulations and extensive replication contributing to their senescence; however, whether the CD8^+^ TEMRA cells contribute to age‐related inflammatory diseases by their elevated proinflammatory functions, remains unknown. We used LIPS approach as it quantitatively assesses the antibody levels and is suitable to detect antibodies directed against linear and conformational epitopes. As LIPS method has a dynamic range of measurement, we were able to use correlation analysis to define CD8^+^ TEMRA as the cell type, which is most associated with anti‐CMV antibody levels. The frequent CMV activation induces antibodies and T‐cell clones to the virus, which are mostly of CD8^+^ EM and TEMRA origin (Fuchs et al., 2019). A recent study, however, showed that the changes in the T‐cell pool of CMV‐infected individuals cannot be explained by the presence of large numbers of CMV‐specific T‐cells suggesting that CMV infection may also affect the phenotype of non‐CMV specific CD8^+^ T‐cells (van den Berg et al., 2021).

We also studied TEMRA cell correlations with inflammation proteins. We found the strongest association with TRANCE/RANKL, a TNF family cytokine well known for its functions in the immune system and bone differentiation (Hanada et al., 2011), which levels correlated negatively with CD8^+^ TEMRA and its subpopulations as well as with CD4^+^ TEMRA and EM subpopulations. TRANCE/RANKL has a role in the regulation of bone metabolism, as well as the formation of lymphoid organs such as thymus, lymph nodes, and Peyer's patches (Sobacchi et al., 2019). In the thymus, TRANCE/RANKL is produced by differentiating CD4^+^ T‐cells and is involved in the differentiation of medullary thymic epithelial cells, which act as mediators of the thymic tolerance process (Rossi et al., 2007). Thus, its lower levels may indicate thymic involution and a decrease in naive T‐cells. TRANCE/RANKL plasma levels have also been reported lower in patients with nonalcoholic fatty liver disease (Nikseresht et al., 2020), a condition related to insulin resistance and obesity. TRANCE/RANKL has been shown to suppress proinflammatory cytokine production in mouse model (Maruyama et al., 2006); however, in humans, the RANKL gene mutations do not result in an increased risk of immune disorders and its inhibitor (denosumab) has no significant effect on inflammatory processes (Ferrari‐Lacraz & Ferrari, 2011). The mechanistic link between the decreased systemic levels of TRANCE/RANKL and increased proportions of TEMRA cells remains to be studied.

CD8^+^ TEMRA population with their reduced capacity to replicate, decreased survival, and high expression of nuclear γH2AX can serve as an indicator of age‐related T‐cell senescence. We, therefore, developed a statistical model for CD8^+^ TEMRA quantification using site‐specific DNA methylation levels. The data of differentially methylated DNA at transcriptionally active chromatin regions have been used to deconvolute major cell types in peripheral blood (Houseman et al., 2012; Koestler et al., 2013), to predict biological (Hannum et al., 2013; Horvath, 2013) and immunological age (Alpert et al., 2019). Epigenetic qPCR assays for analysis of human immune cell populations, including CD4^+^ and CD8^+^ T‐cells, correlated well with flow cytometry and could also be applied to dried blood spots (Baron et al., 2018). We here used bisulfite amplicon sequencing, which can be considered as the gold standard for measuring DNA methylation because of the single‐nucleotide resolution, flexibility, and low input of genomic DNA. The CpG methylation analysis with deep‐amplicon bisulfite sequencing showed the best all‐round performance in multicenter benchmarking study evaluating DNA methylation assays for clinical use (BLUEPRINT‐Consortium, 2016).

In summary, our results support the idea that not the chronological but rather the molecular changes or chronic infections occurring during the aging drive the immune senescence. In this regard, CD8^+^ TEMRA cells can be used as a biomarker to follow the changes associated with immunosenescence. In contrast to flow cytometry, the epigenetic quantification of CD8^+^ TEMRA cells from whole blood DNA is less costly and time‐consuming and can enable a high throughput screening of a large number of samples for better stratification of immunosenescence in old individuals.

## 4 EXPERIMENTAL PROCEDURES

### 4.1 Study subjects

The study was approved by the Ethics Review Committee of Human Research of the University of Tartu according to permissions no 272/T‐12, 275/M‐17, 163/T‐6, and 242/M‐8. Altogether we used samples from 165 individuals (123 females and 42 males) with an age range between 4 and 96 years. Of those, 140 (105 females and 35 males) were over 65 years old and formed the main study group, described in Table 1. From these old individuals of the main cohort, we collected data of flow cytometry results of 26 CD4^+^ and CD8^+^ T‐cell subpopulations, methylation levels of 191 CpG sites, clinical information, levels of 92 inflammation associated proteins, and levels of CMV specific antibodies. The disease information was obtained from the Estonian eHealth system. The disease diagnosis was confirmed by the specialty doctor and marked by the corresponding international classification of diseases code (ICD‐10) in the patient's eHealth records. The remaining 25 individuals, mostly younger people, were studied for DNA methylation (145 CpG sites, a subset of 191 CpG sites) and the proportions of neutrophils, monocytes, NK cells, lymphocytes, T‐cells, CD4^+^ T‐cells, CD8^+^ T‐cells, and CD8^+^ TEMRA cells. The main cohort (old individuals) was used throughout the study while the analysis of the younger group of additional 25 people was included in the analysis of age‐related changes over the entire life span (Figure S1), in calculating the Pearson's correlations between flow cytometry and DNA methylation (Figures S7) and modeling T‐cell, CD8^+^ T‐cell, and CD8^+^ TEMRA cell proportions in respect to WBC (Figure 5 and Figure S9).

### 4.2 PBMC extraction and flow cytometry

The blood samples were collected as a routine sample collection at the admission to the hospital in the morning; however, without the requirement to fast. The blood samples were kept at room temperature and processed within 3–5 h after the collection time. In a few cases, the samples were kept at room temperature overnight and then processed the next morning. We have not found this to affect T‐cell viability or their subset distribution. Peripheral blood mononuclear cells (PBMC) were extracted using Ficoll‐Paque (GE Healthcare, Chicago, IL, USA) gradient centrifugation. Plasma was collected before the extraction and isolated cells were stored using CTL‐Cryo ABC Media Kit (CTL) in a −150°C freezer. Immune cell subtypes were analyzed by flow cytometry (Cossarizza et al., 2019) as reported previously (Oras et al., 2019) using FITC anti‐human CD25 (cat no 303604), PerCP/Cyanine5.5 anti‐human HLA‐DR (cat no 307629), APC anti‐human CD31 (cat no 303115), Alexa Fluor 700 anti‐human CD4 (cat no 317425), Biotin anti‐human CD127 (cat no 351346) + Brilliant Violet 421 Streptavidin (cat no 405225), Brilliant Violet 510 anti‐human CD27 (cat no 302836), Brilliant Violet 605 anti‐human CD279 (cat no 329924), Brilliant Violet 650 anti‐human CD3 (cat no 317324), PE anti‐human CD57 (cat no 359612), PE/Dazzle 594 anti‐human CD197 (cat no 353236), PE/Cyanine5 anti‐human CD28 (cat no 302910), PE/Cyanine7 anti‐human CD45RA (cat no 304126, all from Biolegend), and BUV395 Mouse Anti‐Human CD8 (cat no 563795, BD Biosciences). Cells were acquired with LSR Fortessa flow cytometer (BD Biosciences). Data were analyzed using FCS Express 7 (DeNovo Software). The cells were gated to exclude debris, dead cells, and doublets. The gating strategies are shown in Figure S10.

### 4.3 DNA methylation analyses

Whole blood was collected to K2E (EDTA) vacutainers (Becton Dickinson) and donors’ genomic DNA was extracted from 1 ml of whole blood by the salting‐out method. The DNA sample purity and concentrations were measured by NanoDrop ND‐1000 spectrophotometry. Genomic DNA (500 ng) was treated with sodium bisulfite using the EZ DNA Methylation Kit (Zymo Research Corporation) according to the manufacturer's instructions. Bisulfite treated DNA was amplified in 10 ul reaction containing 0.72 ng of DNA, 1X Yellow PCR Buffer with (NH4)2SO4 (Naxo), 1.5mM MgCl_2_ (Solis BioDyne), 2 mM dNTP mix (Solis BioDyne), 0.2 µM of each primer, and 0.06 U HOT FIREPol DNA Polymerase (Solis BioDyne). Cycle conditions were as follows: 95°C for 15 min, 1 cycle; 40 cycles (95°C for 20 s, 56°C for 30 s, 72°C for 1 min); and 72°C for 3 min, 1 cycle. Primer sequences used in PCR reactions can be delivered on request by the authors. Amplicons from each individual were combined in equal amounts, purified with Agencourt AMPure XP beads (Beckman Coulter) and labeled with Nextera XT v2 (Illumina) indexes. Paired‐end sequencing of bisulfite‐treated DNA with read length of 250 bp was done with Illumina MiSeq at the Core Facility of the Institute of Genomics of the University of Tartu.

### 4.4 CMV antibody analysis with ELISA and LIPS

ELISA kits SmartEIA CMV IgG and EIA CMV IgM (both from TestLine Clinical Diagnostics s.r.o.) were used to measure the IgG and IgM antibodies to CMV with the plasma dilution of 1:101. LIPS profiling of CMV antibodies with pp150 protein fragments has been reported earlier (Burbelo et al., 2009). Two fragments of immunodominant regions of CMV antigen pp150 were cloned into pNanoLuc vector, and LIPS was performed as reported earlier (Haljasmägi et al., 2020). The HEK293 cell lysates containing NanoLuc‐fusion proteins (0.5–1 × 10^6^ luminescence units; LU) were incubated with plasma samples and Protein G Sepharose beads (Creative BioMart) to capture antibodies (in 1:40 dilution). After washing, the substrate was added (Nano‐Glo™ Luciferase Substrate, Promega), and luminescence was measured in VICTOR X Reader (PerkinElmer Life Sciences).

### 4.5 Olink proximity extension profiling

The plasma samples of old individuals were studied using Proseek Multiplex Inflammation panel by Olink Proteomics analyzing 92 inflammation‐related protein biomarkers in total. The assay uses two oligonucleotide‐conjugated antibodies that bind to protein targets and the paired oligonucleotide sequences are amplified by quantitative real‐time PCR reaction. Data are given as normalized protein expression (NPX) values on a log_2_ scale. Proteins containing NPX values >50% below the assay's limit of detection were excluded from the analysis.

### 4.6 Data analysis

Firstly, we assessed the quality of the obtained paired‐end reads with FastQC (version 0.11.5) and MultiQC (version 1.4). Subsequently, we removed adapters and trimmed low‐quality sequences (at quality threshold Phred score 35) with Cutadapt (version 1.18) and TrimGalore (version 0.5.0) correspondingly. The parameters for TrimGalore were the following: ‐‐paired ‐‐quality 35. We aligned the reads to the reference genome (GRCh37/hg19) and extracted site‐specific methylation levels with Bismark (version 0.18.1) in combination with Bowtie 2 aligner (version 2.3.4.1). More precisely, after building the indexed genome with Bismark via using default parameters, we aligned reads again with Bismark now using arguments ‐n 1 ‐X 1000 which was followed by methylation levels extraction with arguments ‐p ‐‐no_overlap ‐‐bedGraph ‐‐counts ‐‐buffer_size 10G.

Subsequently, we aggregated coverage files into a single dataset, where we filtered out methylation values that had read depth <300 to be confident in the obtained methylation levels. Overall read depth was in the range of 1000–5000. After filtering, we excluded CpG sites that had missing values for many individuals, which was done to such an extent that the resulting dataset contained less than 5% of missing values overall. Then, we imputed those missing values with R package missForest (version 1.4) with default parameters. We also visualized the imputation accuracy via plotting low coverage values against imputed counterparts that is shown in Figure S11. As expected, there was a good agreement between imputed and actual methylation levels (r = 0.96) with methylation levels that came from smaller read depth deviating further from the predicted values.

We used R (version 4.0.2) for data analysis. More precisely, for data pre‐processing, we used packages dplyr (version 1.0.5) and reshape2 (version 1.4.4). For visualization, we used ggplot2 (version 3.3.3), ggpubr (0.4.0), scales (version 1.1.1), rstatix (version 0.7.0), and ComplexHeatmap (2.7.1). Eventually, we combined individual plots with patchwork (version 1.1.1). Overall in the analysis, we determined the statistical significance for group‐wise comparisons using either two‐sample Wilcoxon test (also known as Mann‐Whitney test) or linear regression where age and gender were controlled. The methods used for each particular comparison is specified in the corresponding figure legend. We used either Pearson's or Spearman's correlation coefficients depending on the distribution of the measurements and the nature of the relationship. In addition, where suitable, partial correlations were calculated with package ppcor (version 1.1), in order to control for the age effect. All *p*‐values shown on the figures are adjusted with FDR method.

To model flow cytometry measurements based on DNA methylation, we relied mostly on Pearson's correlations and linear models since a linear relationship could be assumed. Specifically, we selected the CpG sites so that they would be specific to a certain cellular population and therefore the studied sites’ methylation levels should be proportional with the fraction of the cell's subset. Since the number of CpG sites was significantly greater than sample size / 10, we applied multiple feature selection methods in a sequence to keep only a small number of relevant CpG sites for the modeling task. Firstly, we split the dataset into training and test set via R package Caret that we also used for parameter tuning and training all the models. In addition, since many flow cytometry measurements that we used as dependent variables were deviating from a normal distribution (based on visual inspection on quantile‐quantile plots and those whose Shapiro‐Wilk test *p*‐value <0.05), we transformed those measurements so that their distributions came closer to normal distribution. We used square root and cube root to transform measurements “CD8^+^ T‐cells/WBC” and “CD8^+^ TEMRA cells/WBC” correspondingly. In those cases, the predictions of those models were inverse transformed for further analysis and visualization. After transformation, we obtained a set of relevant features for each dependent variable via Boruta feature selection method (Kursa & Rudnicki, 2010) on training data. We then reduced the number of CpG‐s further via lasso regression on previously obtained features. Subsequently, we evaluated the models with a nested 5‐fold cross‐validation scheme, using the inner folds for hyperparameter tuning (in the case of the random forest the hyper parameter was the number of trees and in the case of ridge regression it was lambda). We used RMSE to evaluate the model's performance. Finally, we tested all models on the test dataset and visualized their predictions in comparison with their actual values.

## CONFLICT OF INTEREST

The authors declare no conflict of interests.

## AUTHOR CONTRIBUTION

AS performed the bioinformatics and statistical data analyses, and epigenetic model development. LT designed the experiments and performed DNA methylation and amplicon sequencing. LT, KK, and AO did flow cytometry analyses. APM did CMV antibody analyses. LT was involved in proximity extension analyses. KKingo, KS and RT were responsible for the collection and documentation of blood donor samples. RU, HP, KKisand and PP supervised, designed and were overall responsible for the study. AS and PP wrote the manuscript.

## Supporting information

Fig S1

Fig S2

Fig S3

Fig S4

Fig S5

Fig S6

Fig S7

Fig S8

Fig S9

Fig S10

Fig S11

Table S1‐S14

## Data Availability

The data supporting the conclusions of this article will be made available by the authors, without undue reservation.
