# Supplementary figures and images for: Epigenetic quantification of immunosenescent CD8+ TEMRA cells in human blood
Source: Aging Cell. 2022 Apr 9;21(5):e13607. doi: 10.1111/acel.13607 (PMC9124311; doi:10.1111/acel.13607)

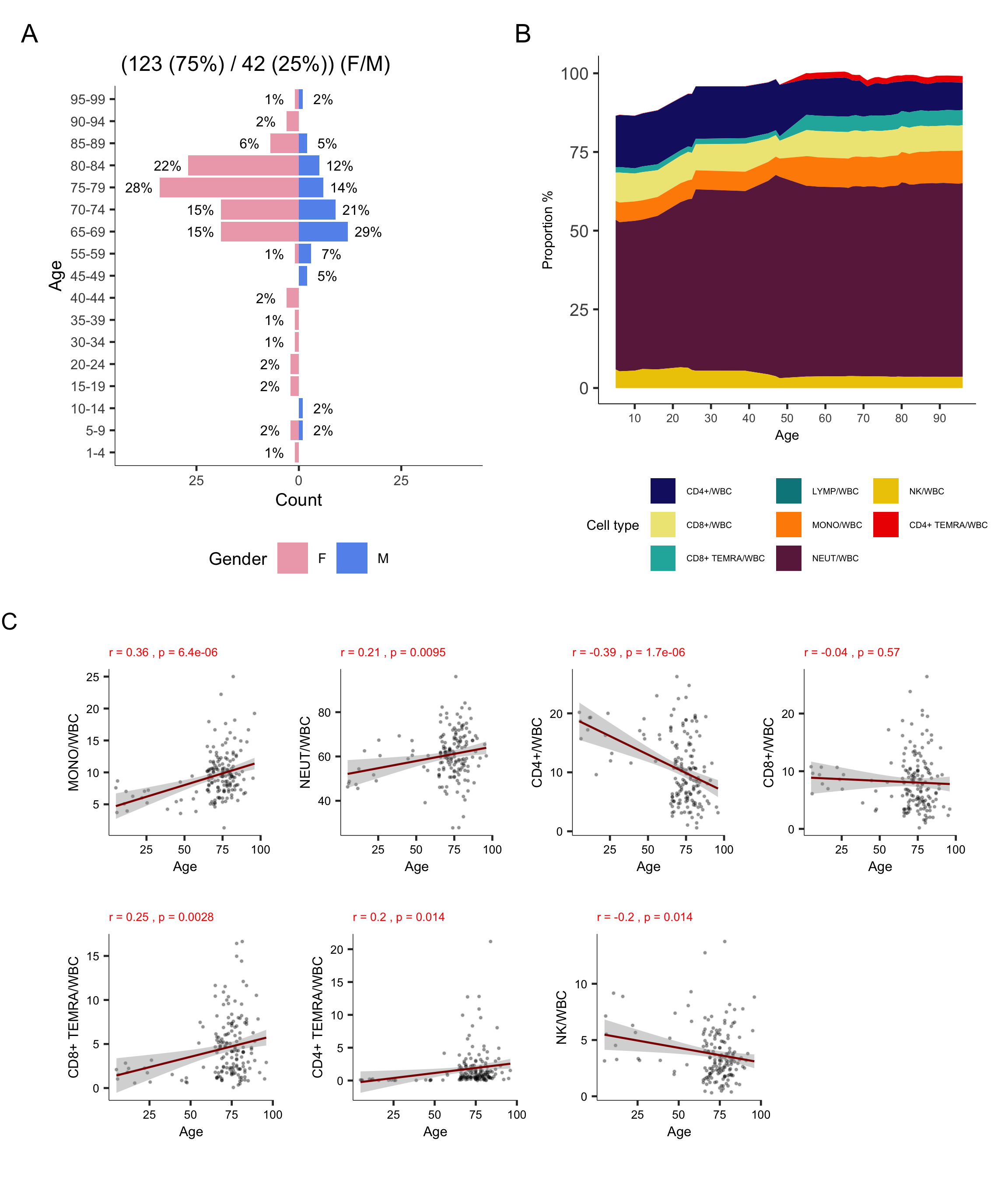

Supplement: Supplementary file 1 — Fig S1 [file ACEL-21-e13607-s006.png]

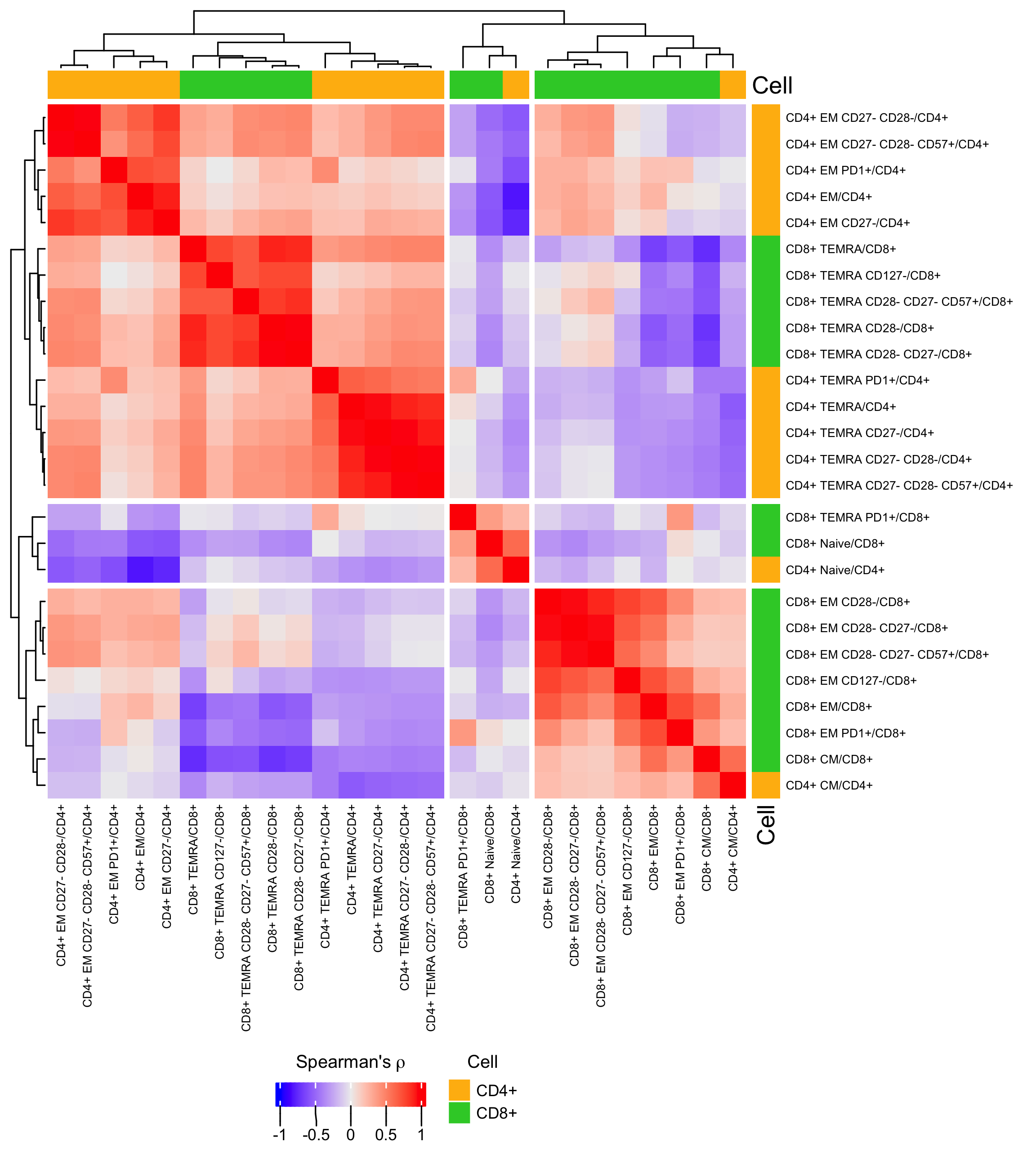

Supplement: Supplementary file 2 — Fig S2 [file ACEL-21-e13607-s005.png]

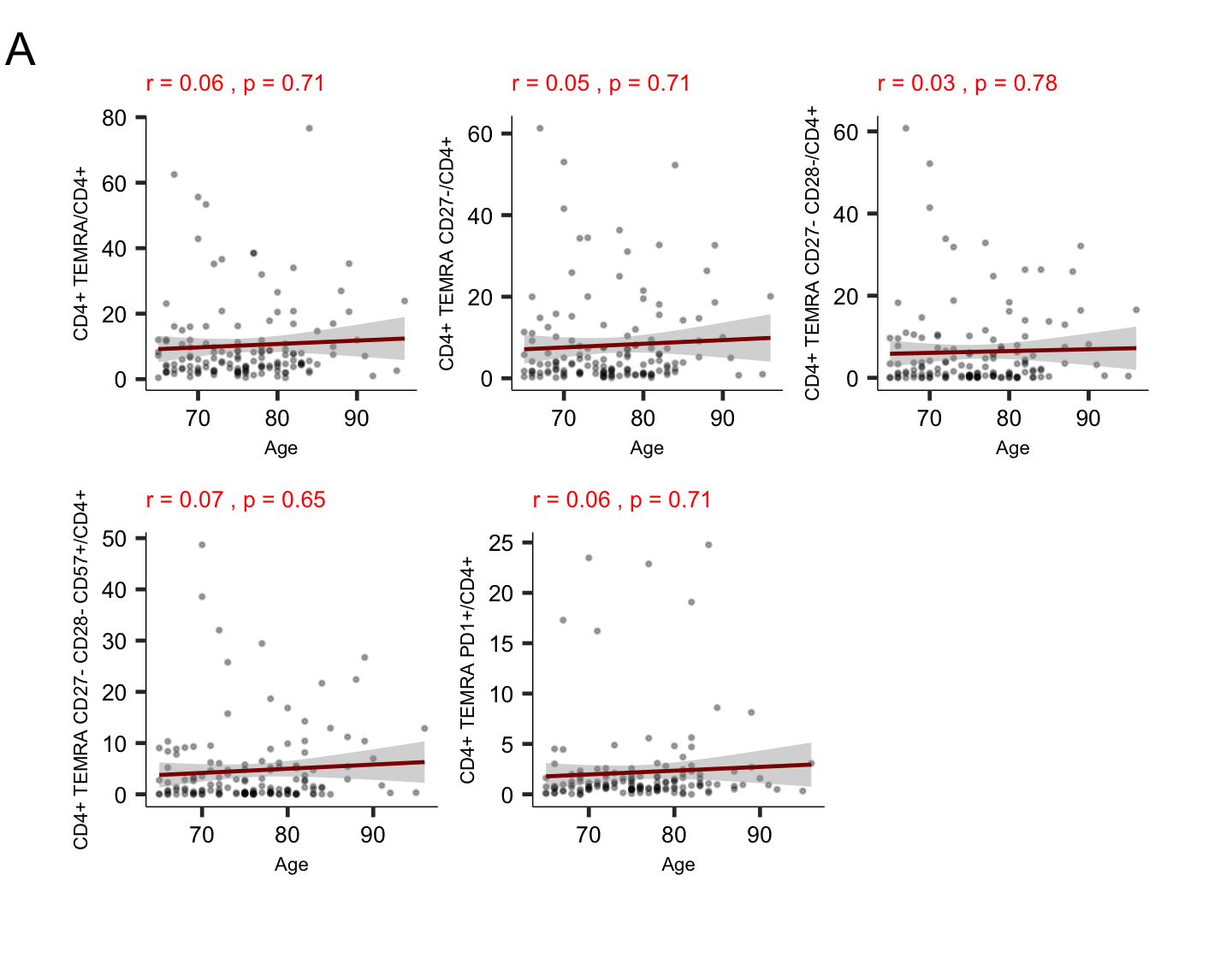

Supplement: Supplementary file 3 — Fig S3 [file ACEL-21-e13607-s012.png]

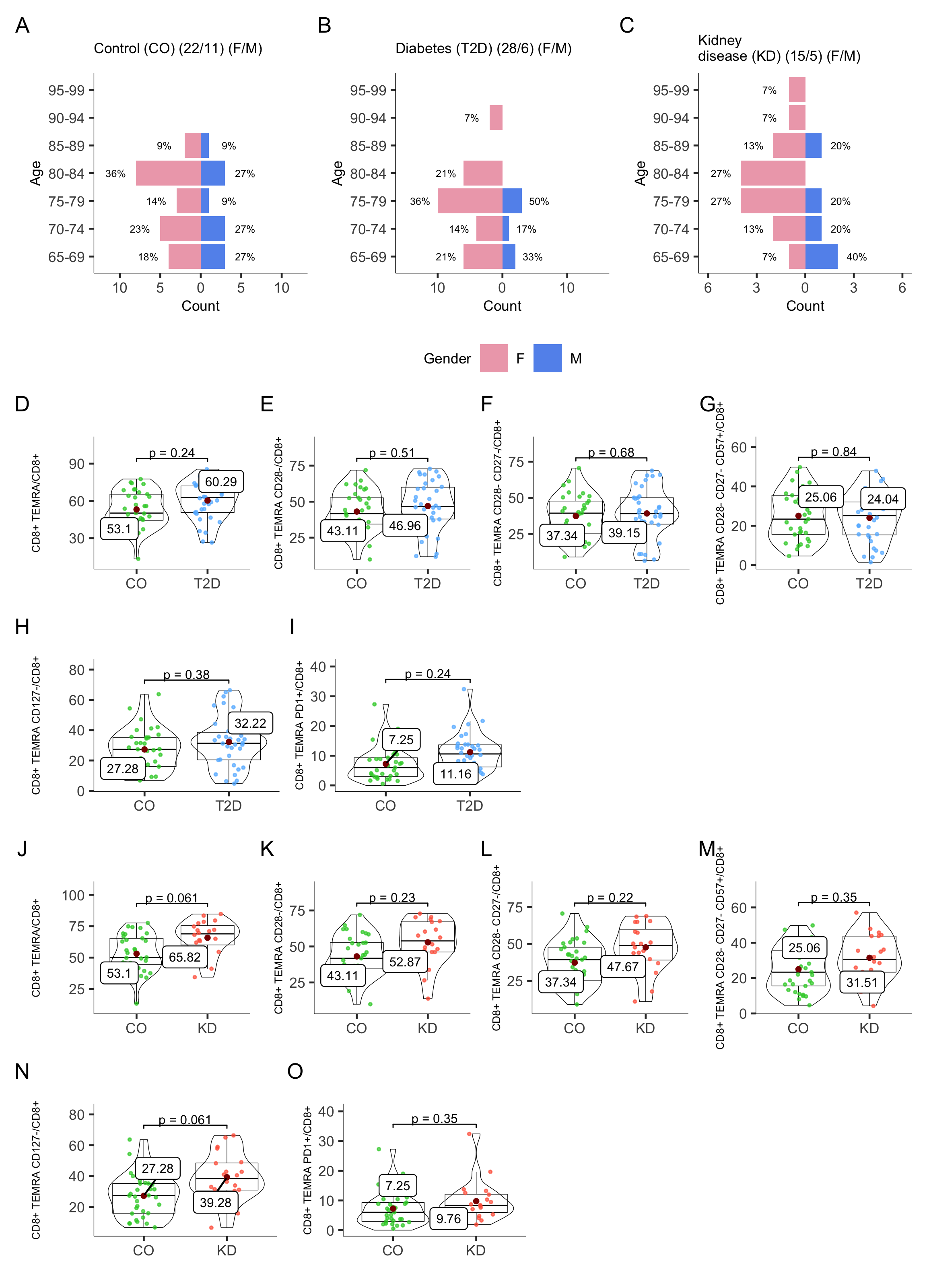

Supplement: Supplementary file 4 — Fig S4 [file ACEL-21-e13607-s011.png]

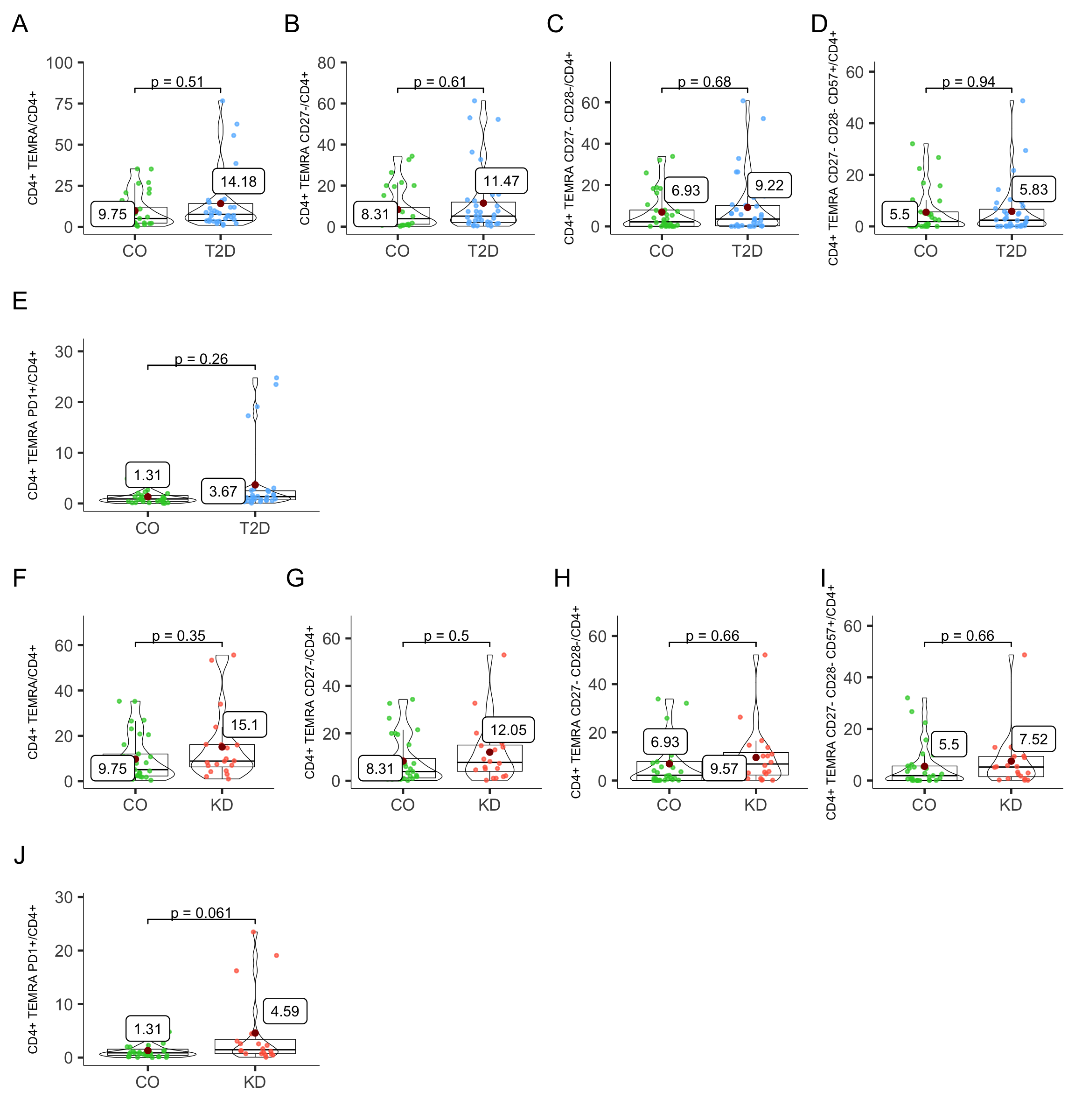

Supplement: Supplementary file 5 — Fig S5 [file ACEL-21-e13607-s008.png]

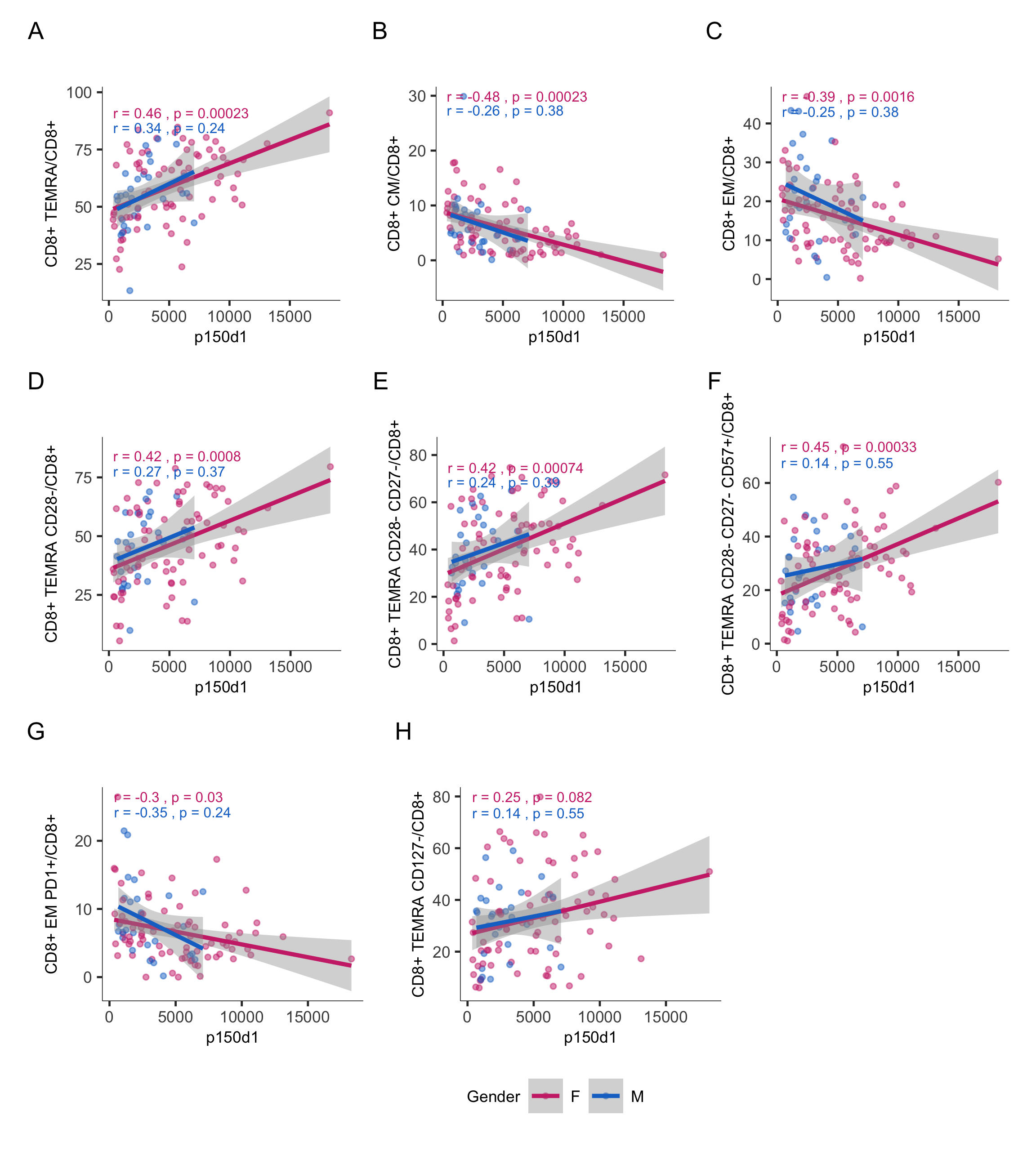

Supplement: Supplementary file 6 — Fig S6 [file ACEL-21-e13607-s004.png]

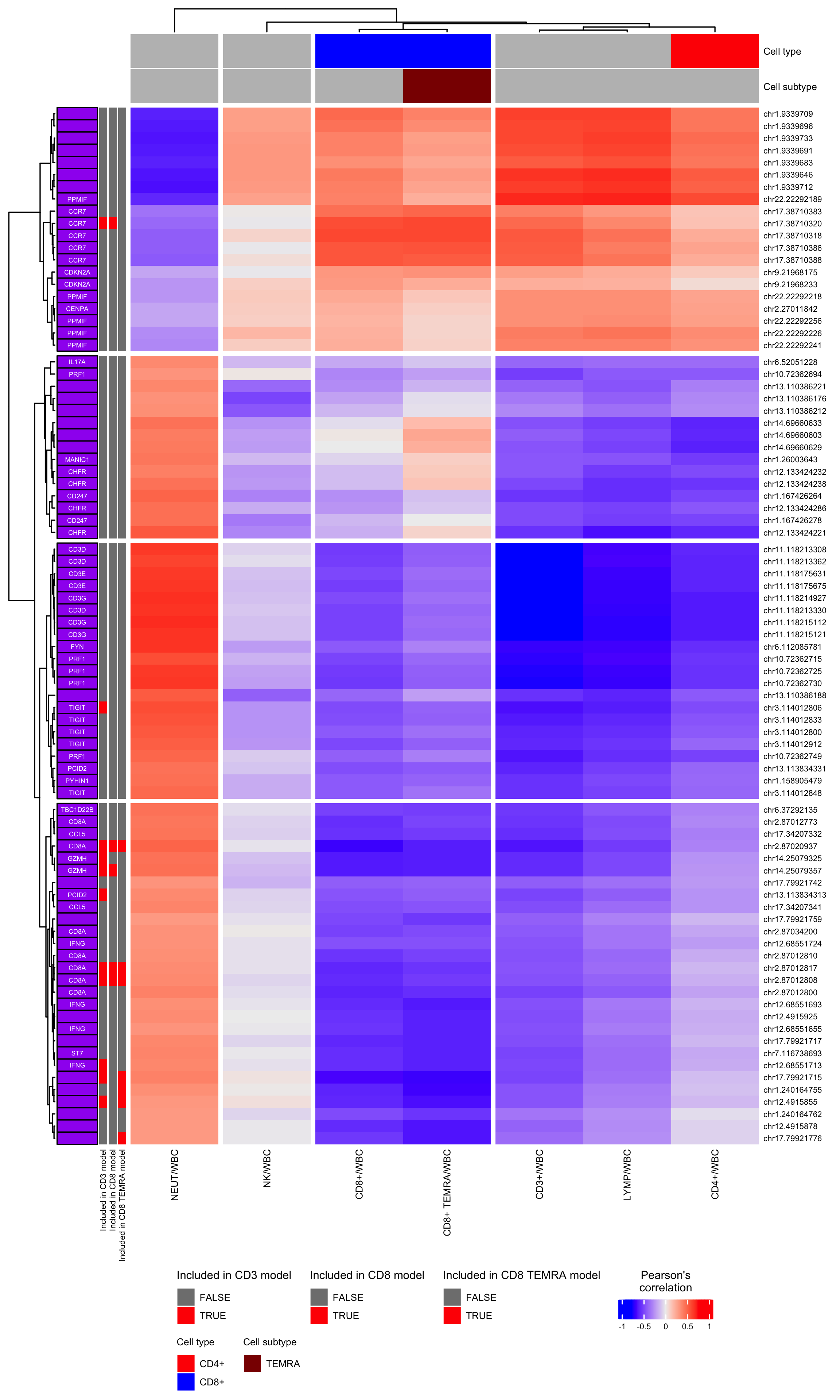

Supplement: Supplementary file 7 — Fig S7 [file ACEL-21-e13607-s010.png]

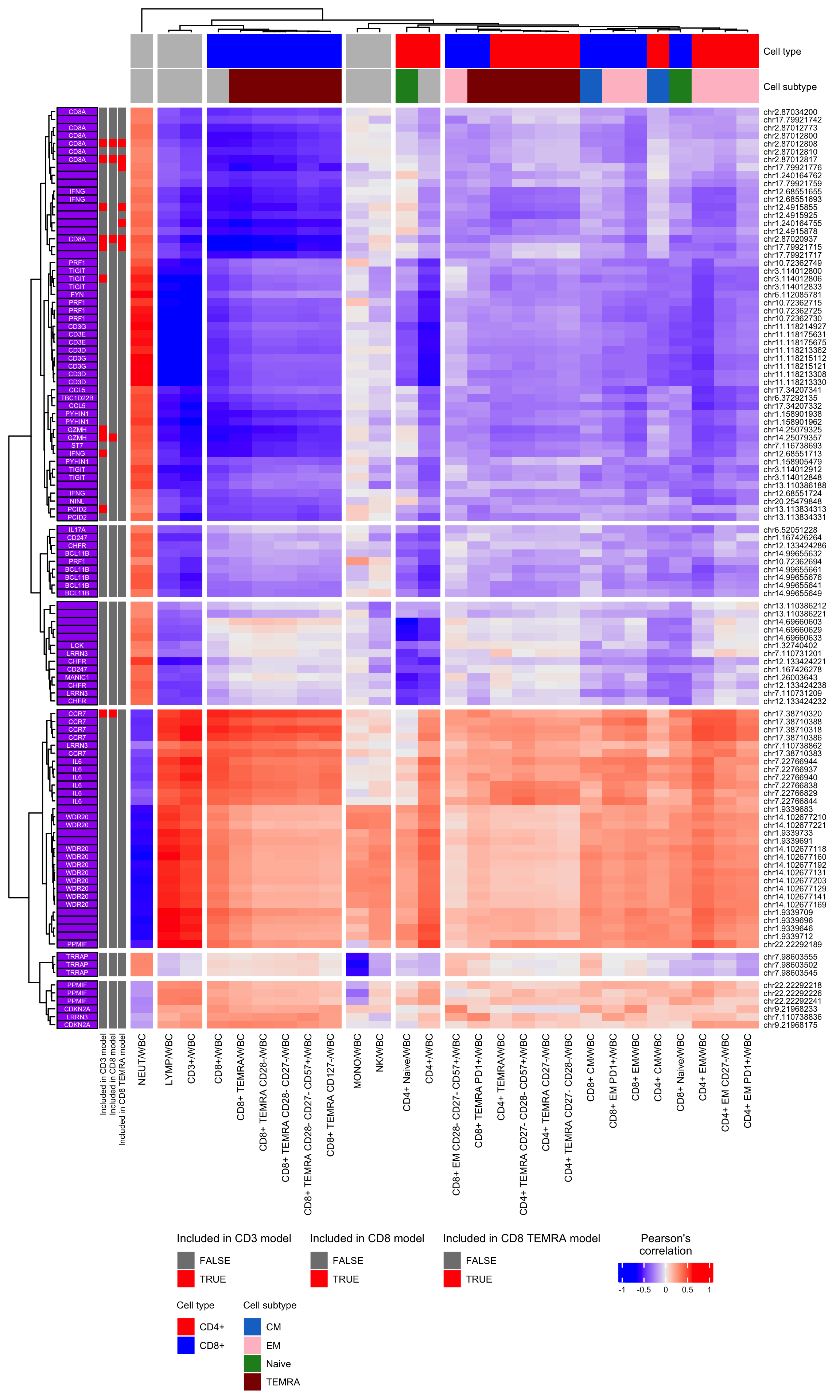

Supplement: Supplementary file 8 — Fig S8 [file ACEL-21-e13607-s009.png]

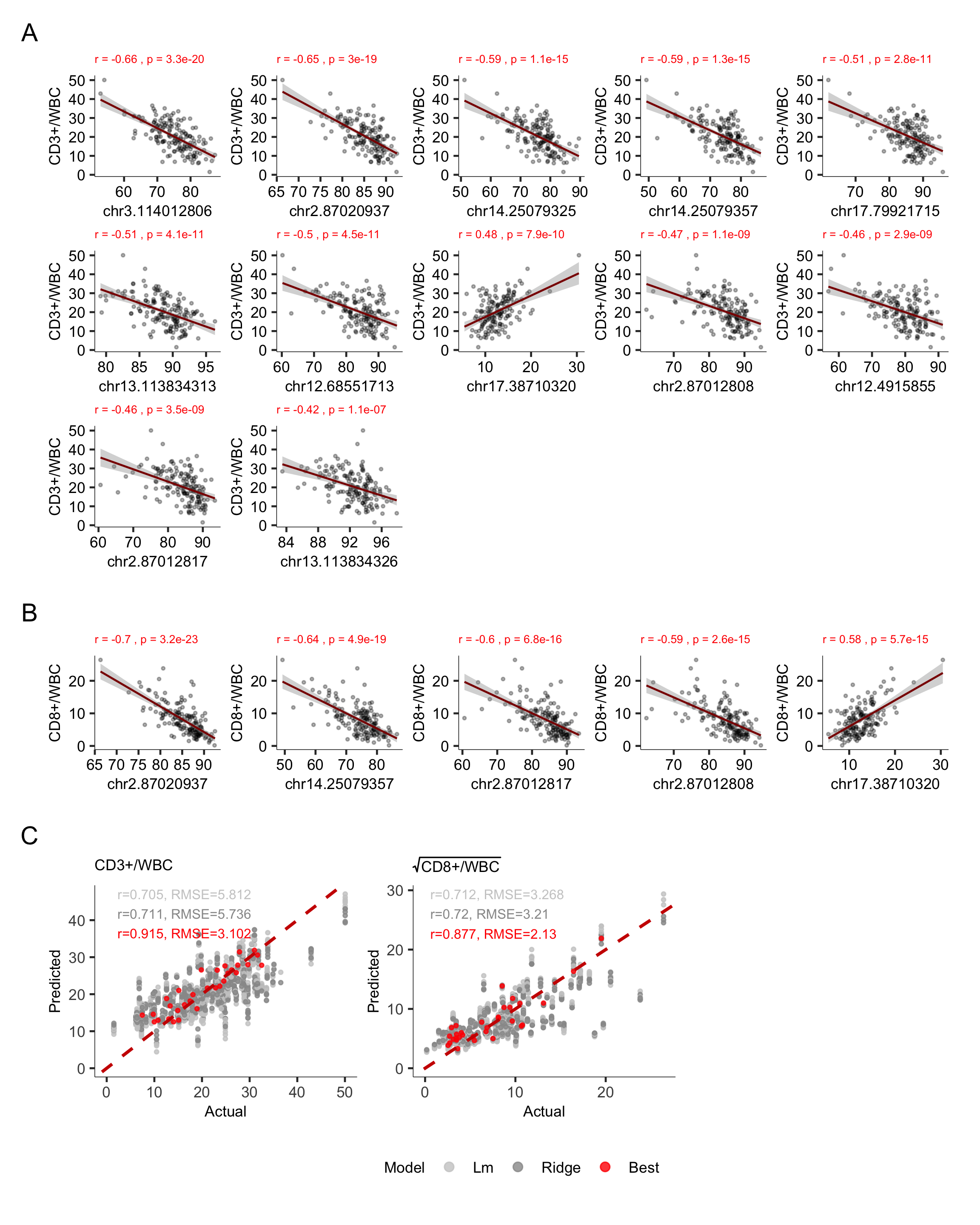

Supplement: Supplementary file 9 — Fig S9 [file ACEL-21-e13607-s002.png]

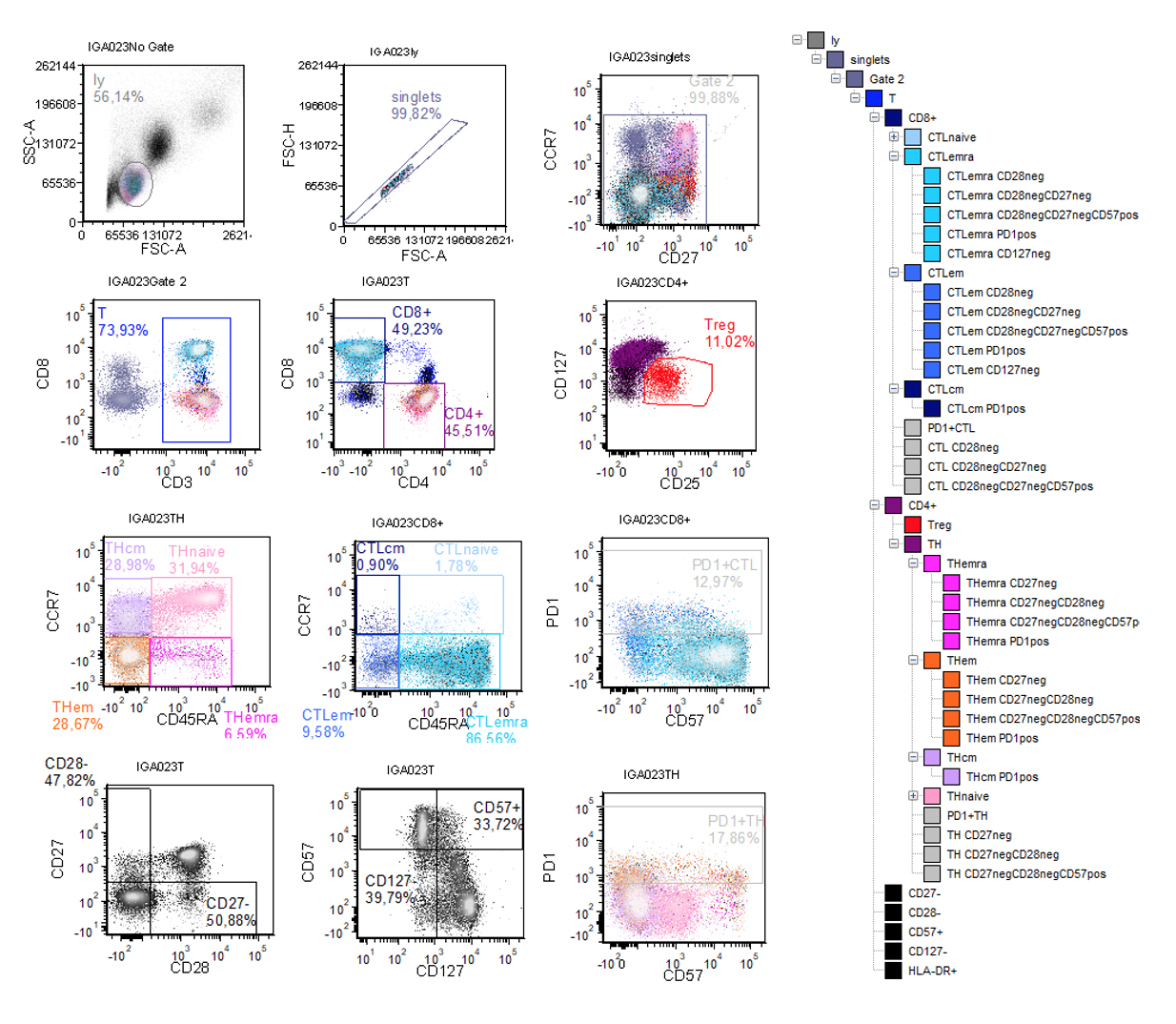

Supplement: Supplementary file 10 — Fig S10 [file ACEL-21-e13607-s003.png]

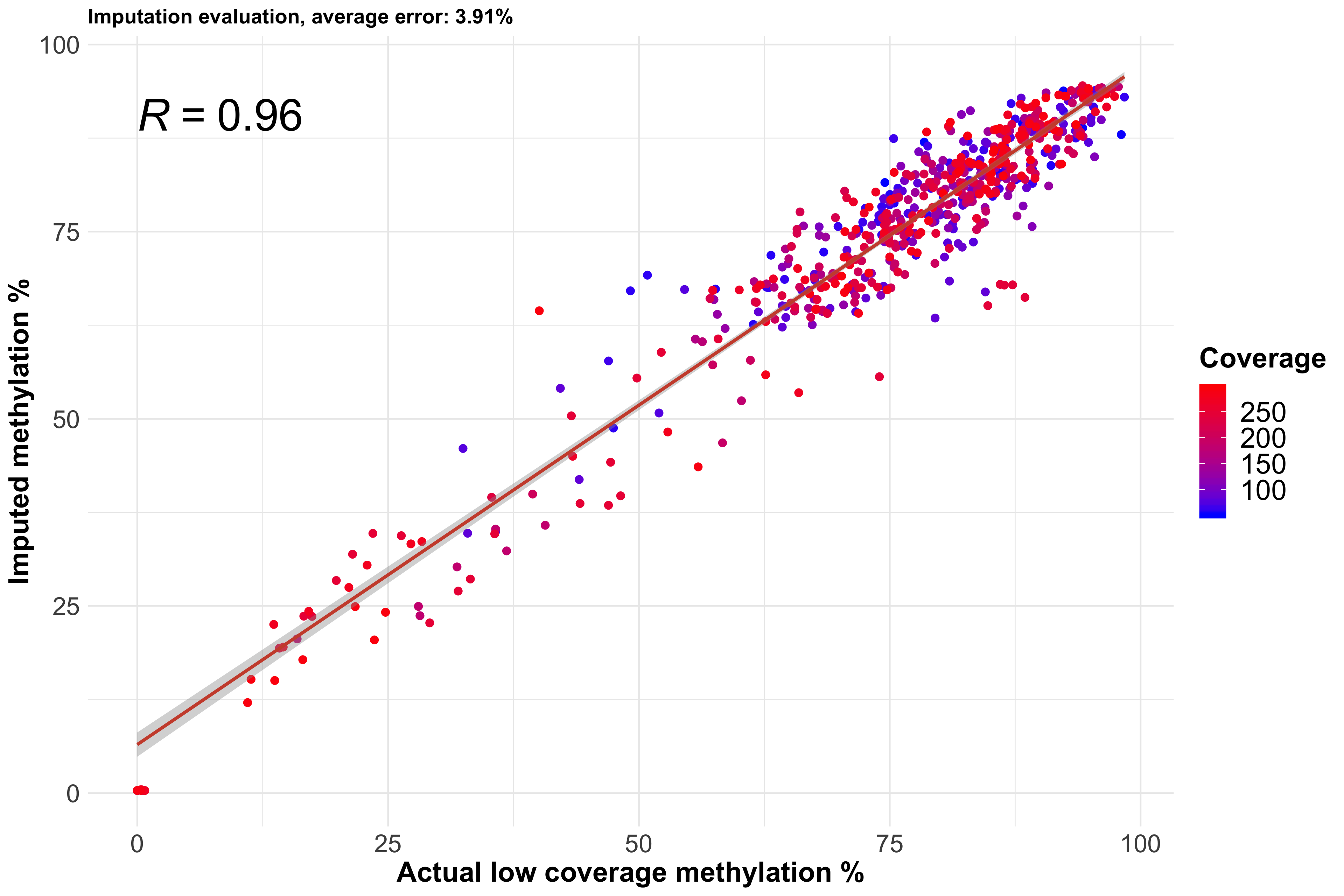

Supplement: Supplementary file 11 — Fig S11 [file ACEL-21-e13607-s007.png]
